# Identification of linear B-cell epitopes of Senecavirus A VP2 protein using monoclonal antibodies

**DOI:** 10.3389/fmicb.2025.1546925

**Published:** 2025-03-05

**Authors:** Yao Jiang, Zhenhua Guo, Maoyang Weng, Linlin Chen, Qingmei Li, Lei Zhang, Songlin Qiao, Gaiping Zhang

**Affiliations:** ^1^College of Veterinary Medicine, Northwest A&F University, Yangling, Shaanxi, China; ^2^Key Laboratory of Animal Immunology of the Ministry of Agriculture, Institute for Animal Health, Henan Academy of Agricultural Sciences, Zhengzhou, China; ^3^Ministry of Agriculture and Rural Affairs Key Laboratory of Veterinary Biological Products and Chemicals, China Animal Husbandry Industry Co., Ltd., Beijing, China; ^4^School of Advanced Agricultural Sciences, Peking University, Beijing, China; ^5^Longhu Laboratory, Zhengzhou, Henan, China

**Keywords:** Senecavirus A, VP2 protein, monoclonal antibodies, B-cell epitopes, spatial structure

## Abstract

**Introduction:**

Senecavirus A (SVA) is an emerging vesicular pathogen in swine with clinical signs similar to those of foot-and-mouth disease, porcine vesicular disease and vesicular stomatitis, making the control of swine vesicular disease outbreaks difficult. Therefore, the development of effective diagnostics and vaccines for SVA has become critical. VP2 is a structural protein that elicits a strong immune response, which positions it a candidate for diagnostic and vaccine development.

**Methods:**

In this study, five high-titer monoclonal antibodies (mAbs) were produced using hybridoma technology. Twenty-eight peptides covering the entire VP2 sequence were synthesised by overlapping peptide synthesis, and the positive peptides were screened with the five mAbs by ELISA and Dot-blotting. The peptides were then further truncated to identify the minimal epitope regions based on immunoinformatics analyses.

**Results:**

Four mAbs were identified that reacted with peptide 15 and one mAb reacted with peptide 26. Further truncation of these peptides led to the identification of two novel minimal epitopes: 156-NEEQWV-161 and 262-VRPTSPYFN-270. Structural and sequence alignment analyses revealed that epitope 156-NEEQWV-161 is located in the flex-loop region of the VP2, whereas epitope 262-VRPTSPYFN-270 is located in the β-sheet of the VP2. Both epitopes were highly conserved among typical SVA isolates from different countries.

**Discussion:**

This study identifies two novel B-cell epitopes on the VP2, contributing to the development of VP2-based diagnostic tools with clinical applications. The findings also provide valuable material for the design of novel vaccines against SVA, offering new insights into the immune response to this pathogen.

## 1 Introduction

SVA was first isolated from the contaminant of the PER.C6 cell line and named SVV-001 in 2002 (Hales et al., 2008). Since SVA does not infect normal human tissues but specifically targets neuroendocrine cells, early studies focused on it as a natural oncolytic virus (Segalés et al., 2017). The pathogenicity of the virus was unclear until 2007, when vesicular lesions were detected in pigs in Canada and confirmed as SVA infection by reverse transcription PCR and virus isolation (Pasma et al., 2008). Before 2014, SVA was infrequently associated with porcine vesicular disease. Since the outbreak of SVA-related vesicular disease in Brazil in 2014, cases have been reported in the United States (Canning et al., 2016), Canada (Xu et al., 2017), China (Wang et al., 2020), Vietnam (Arzt et al., 2019), Colombia (Sun et al., 2017), Thailand (Saeng-Chuto et al., 2018), and other countries. The reemerging SVA strains shared about 94% nucleotide identity with the prototype strain SVV-001 (Guo et al., 2020). These new prevalent strains of SVA show increased pathogenicity, causing severe blister-like lesions in the nasal cavity, mouth and coronary band of the hoof. Infected pigs present with fever, lameness, and lethargy with rapid spread (Leme et al., 2017; Zhang et al., 2018). It has been reported that neonatal piglets under 7 days of age occasionally suffer from diarrhoea with high mortality rates (Vannucci et al., 2015). With the spread of SVA, viral mutations have accelerated, threatening the stability of the global swine industry. Rapid and accurate diagnosis is essential to differentiate SVA from other clinically similar vesicular diseases and to implement timely control measures.

SVA particles are round, non-enveloped, and icosahedrally symmetric (Venkataraman et al., 2008). The genome is a linear, single-stranded positive-sense RNA of approximately 7.2 kb in length, containing a 5’ UTR, a 3’ UTR and an open reading frame (Liu et al., 2020). It encodes four structural proteins—VP4, VP2, VP3 and VP1—which together form the viral capsid. VP1, VP2, and VP3 share a similar folding arrangement on the surface of viral capsid, while the smaller VP4 is located internally on the capsid (Wang et al., 2023). VP2 interacts with VP1, VP3 and cell receptors to induce SVA infection. It also contains the largest number of epitopes among the structural proteins and is more immunogenic than VP1 and VP3. This makes VP2 an ideal target for diagnostic assays and vaccine development.

Antigenic epitopes are specific amino acid sequences or regions by which the immune system recognises antigens. B-cell epitopes are molecular regions of proteins that are specifically recognised by antibodies. Since the immune response induced by SVA infection is closely linked to the VP2-specific antibody response, it is necessary to research the B-cell epitopes of the VP2 protein (Maggioli et al., 2018). In this study, we successfully generated five mAbs through hybridoma technology. Twenty-eight peptides were synthesized using overlapping peptide synthesis, and two positive peptides were identified by screening with these mAbs. The positive peptides were analysed using to the immunoinformatics software and further truncated to finally obtain the minimal epitopes. These results improve our understanding of the VP2 structure and provide valuable material for the development of new diagnostics and the design of novel epitope-based vaccines for SVA.

## 2 Materials and methods

### 2.1 Cells, viruses, and animals

Porcine kidney-15 (PK-15) cells were purchased from ATCC (Manassas, United States), and BL21 (DE3) competent cells were obtained from TransGen Biotech (Beijing, China). Murine myeloma SP2/0 cells were kindly provided by the Institute for Animal Health, United Kingdom. BL21 (DE3) competent cells were obtained from TransGen Biotech (Beijing, China). SVA strain HeNNY-1/2018 (GenBank accession MK357116) was isolated and maintained in our laboratory. Female BALB/c mice were obtained from Henan Sikebesi-Biological (Zhengzhou, China), and housed in sterile animal facilities according to relevant ethical guidelines.

### 2.2 Expression and purification of VP2

*VP2* was synthesized based on the SVA HeNNY-1/2018 strain and separately inserted into plasmids pET-28a and pGEX-4 T-1 to construct the recombinant expression vectors (Genewiz, China). Verified recombinant plasmids were transformed into BL21 (DE3) competent cells to express His-VP2 and GST-VP2 fusion proteins in the *E. coli* prokaryotic expression system, with empty vectors pET-28a and pGEX-4 T-1 as controls. According to the instructions, the Ni-affinity chromatography column (Smart-Lifesciences, China) was used to purify the soluble fraction of His-VP2. Subsequently, the soluble fraction of GST-VP2 protein was purified according to the GSTPur Glutathione Kit instructions (Smart-Lifesciences, China). Finally, His-VP2 and GST-VP2 were analysed by SDS-PAGE and Western blotting. The protein concentrations of His-VP2 and GST-VP2 were measured using the bicinchoninic acid assays.

### 2.3 Animal immunisation and hybridoma cells production

Five 7-week-old female BALB/c mice were immunised with purified His-VP2. For the first immunisation, 25 μg of His-VP2 was emulsified with an equal volume of Freund’s complete adjuvant (Sigma, United States) to form an oil-in-water emulsion. Then each mouse was injected subcutaneously in scattered spots on the back of the neck. Booster immunisations were given every 2 weeks, twice in total. For subsequent immunisations, we emulsified His-VP2 with Freund’s incomplete adjuvant (Sigma, American), following the same procedures as the first immunisation. One week after the third immunisation, the mice with the highest serum titer tested by indirect ELISA were selected for cell fusion to produce hybridoma cells. Three weeks later, 37.5 μg of purified His-VP2 was diluted in sterile PBS and immunised by intraperitoneal injection as a booster immunisation.

Four days after the intraperitoneal injection, orbital blood was collected and spleen cells were prepared. SP2/0 cells in good growth state were mixed with spleen cells and washed with Glutamic acid, Sodium aspartate, and Potassium chloride (GNK) buffer solution. After the mixed cells were gently knocked apart, 1 mL PEG 1500 (Roche, Germany) was slowly added dropwise and within 1 min. After an additional wash with GNK buffer solution, the fusion cells were suspended in HAT medium, mixed well and seeded into 96-well plates. Five days after cell fusion, clusters of hybridoma cells appeared and positive hybridoma cells were screened by ELISA for secreted high-potency antibodies. Finally, the screened cells were subcloned using a limited dilution method, and the positive rate was still 100% after repeated subcloning three times.

### 2.4 Preparation and characterization of the mAbs

Ascites fluid was produced in female BALB/c mice using the *in vivo* induction method. Each healthy female BALB/c mouse was injected intraperitoneally with 500 μL of sterilized liquid paraffin. Five days later, approximately 1 × 10^6^ hybridoma cells were injected into the peritoneum of a mouse. One week later, abdominal distension due to ascites accumulation was observed and the ascites was collected with a 16 gauge sterile needle. The collected ascites was centrifuged at 500 × g for 15 min. The supernatant was then aliquoted and stored at −80°C. The mAbs were tested for reactivity with VP2, and the mAbs subtype identification kit (Proteintech, China) was used to determine the immunoglobulin class of the mAbs.

### 2.5 Western blotting and indirect ELISA

Western blotting was used to verify VP2 expression and to assess the specificity of the monoclonal antibodies. Protein samples were first run on a polyacrylamide gel for electrophoresis. Proteins isolated after SDS-PAGE were wet transferred to PVDF membranes and immersed in 5% skimmed milk and blocked for 2 h at room temperature (RT). The membranes were incubated overnight at 4°C with either anti-His antibody (Proteintech, China), anti-GST antibody (Proteintech, China), or the previously screened monoclonal antibodies, each at a 1:1000 dilution. Following five washes with PBST (1 × PBS with 0.05% Tween 20, pH = 7.4), the membranes were incubated with HRP-conjugated goat anti-mouse IgG (1:10,000 dilution, Abbkine, United States) for 1.5 h at RT. The PVDF membranes were washed again and treated with NcmECL Ultra (NCM Biotech, China), and the signals were captured using a chemiluminescence imaging system.

As reported previously, indirect ELISA was used to screen positive hybridoma cells and to detect antibody titers. GST-VP2 protein was diluted to 0.5 μg/mL in ELISA coating buffer (Solarbio, China) and coated on a 96-well enzyme plate (100 μL/ well) overnight at 4°C. The plate was washed five times with PBST and then blocked with 5% skimmed milk (200 μL/well) for 2 h at 37°C. Monoclonal antibody samples (100 μL/well) were placed in the wells and incubated at 37°C for 30 min. Then, each well was incubated with 100 μL of goat anti-mouse IgG (1:1000 dilution) at 37°C for 1 h. After five PBST washes, the plate was incubated with TMB one-component substrate solution (100 μL/well) for 10 min at RT, protected from light. The reaction was terminated with 100 μL of 2 M H_2_SO_4_, and absorbance was measured immediately at 450 nm.

### 2.6 Immunofluorescence assay

The specificity of the reaction between anti-VP2 mAbs and SVA was detected using an indirect immunofluorescence assay (IFA). SVA was inoculated into the cells at a multiplicity of infection of 1 and the cells were incubated at 37°C in 5% CO2 for 1 h. When the cytopathic effect reached 75%, the cells fixed with 4% paraformaldehyde (300 μL/well) for 15 min at RT. The cells were then permeabilised with 0.1% Triton X-100 (300 μL/well) for 20 min at RT, followed by blocking with 5% BSA at 37°C for 1 h. Subsequently, the anti-VP2 mAbs (1:1000 dilution) were added and incubated for 1 h at 37°C, followed by Alexa Fluor 488 goat anti-mouse antibody (Invitrogen, United States) under the same conditions. The nuclei were stained with DAPI (300 μL/well, Solarbio, China) for 5 min without light. The cells were then observed under a fluorescence microscope, and images were captured.

### 2.7 Peptide design and synthesis

Based on the overlapping peptide synthesis (Jia et al., 2022), 28 peptides (GL Biochem, China) consisting of 15 amino acids with a 5-amino acid offset were designed. Subsequently, regions with high antigenicity, hydrophilicity, and surface accessibility were selected to further truncate peptides 15 and 26. A cysteine residue was added to the N-terminus of each truncated peptide. The purity of all peptides was above 95%.

### 2.8 Peptide ELISA and Dot-blotting

Peptide ELISA and Dot-blotting were used to screen for specific antigenic regions of anti-VP2 (Shang et al., 2009). Each N-terminal peptide with a cysteine residue was conjugated to BSA (Solarbio, China).

In the peptide ELISA, peptide-BSA conjugate was diluted to 5 μg/mL with ELISA coating buffer (Solarbio, China) and the diluted conjugate (100 μL/well) was coated into a 96-well enzyme plate at 4°C overnight. After washing with PBST, the wells were blocked with 5% skimmed milk at 37°C for 2 h. The next steps were analogous to indirect ELISA.

One microgram of each peptide-BSA was applied to nitrocellulose membranes (Solarbio, China) with GST-VP2 as the positive control and BSA as the negative control. The membranes were blocked with 5% skimmed milk overnight at 4°C. The mAbs (1:1000 dilution) were incubated for 2 h at RT. After 5 washes with PBST, the membranes were incubated with HRP-conjugated goat anti-mouse IgG (1:10,000 dilution) for 1.5 h at RT. Then, the membranes were color developed with NcmECL Ultra (NCM Biotech, China).

### 2.9 Immunoinformatics analysis

The epitope parameters of the peptide 15 and peptide 26 were analysed using Protean (DNAStar, American), respectively. The possible positive epitope regions were selected based on their secondary structure, hydrophilicity, surface accessibility and antigenicity. VP2 sequences from thirty-two SVA strains (Supplementary Table S1) were downloaded from the National Center for Biotechnology Information, aligned using DNAman, and the epitopes were analysed for conservation. The structure of SVA (PDB ID: 6ADT) was obtained from the PDB database, and other protein structures were removed to isolate the VP2 structure. Subsequently, the three-dimensional structure of VP2 protein was visualised using PyMOL to show the spatial arrangement of the VP2 epitopes.

### 2.10 Statistical analysis

In these studies, bands from SDS-PAGE, Western blotting, and Dot-blotting were analysed using ImageJ (1.51j8). Dilutions of the monoclonal antibodies screened were analysed and plotted using Origin (2022). Other data processing and image rendering were performed using GraphPad Prism 8.0.2. The fasta file for VP2 protein was obtained from the PDB database.^1^ Overlapping peptide maps were generated using IBS-Online (Liu et al., 2015).

## 3 Results

### 3.1 Expression and purification of VP2 protein

**Figure 1:**
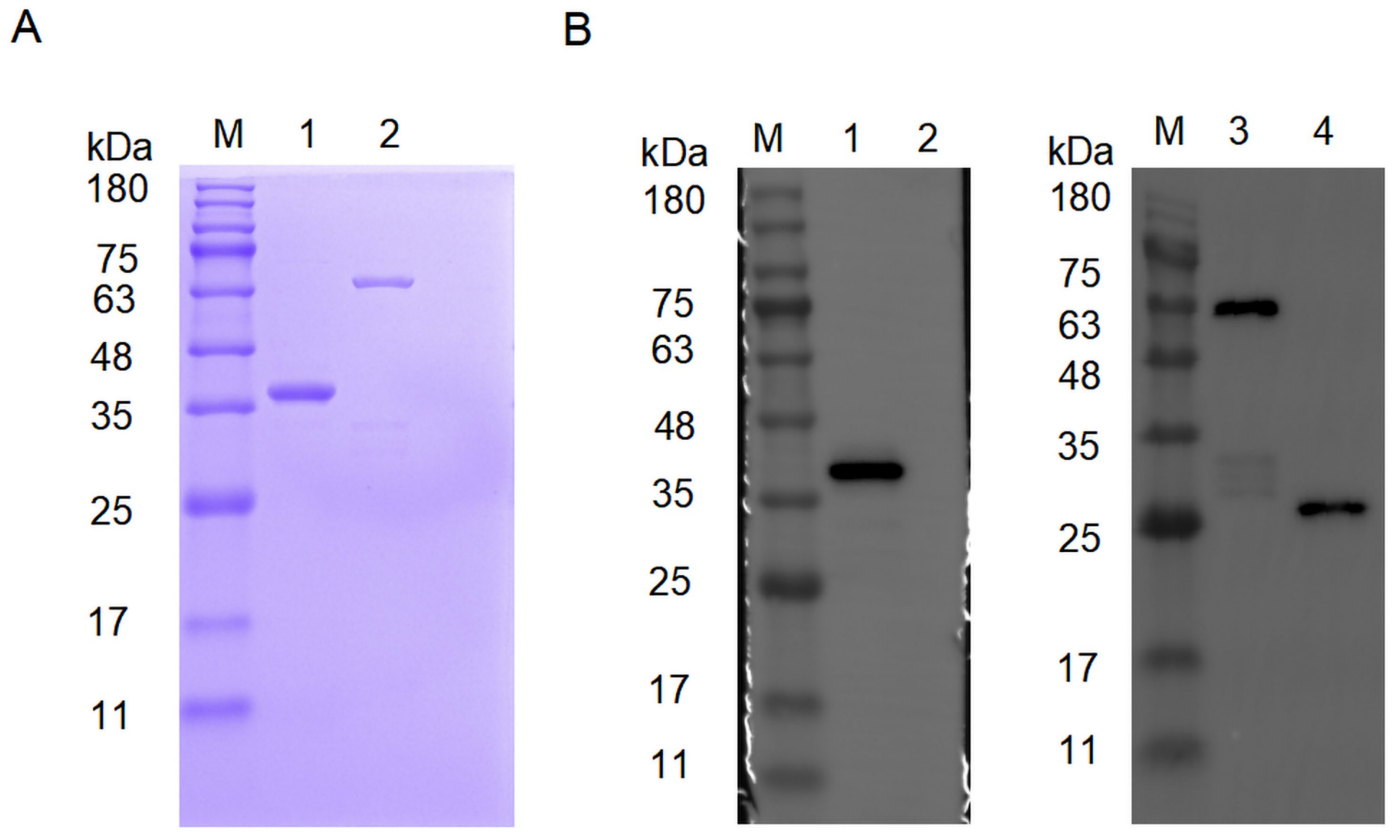
Characterization of recombinant VP2 protein. **(A)** Characterization of His-VP2 protein and GST-VP2 protein by SDS-PAGE. Lane M, protein marker. Lane 1, His-VP2 protein. Lane 2, GST-VP2 protein. **(B)** Characterization of His-VP2 protein and GST-VP2 protein by Western Blotting. The primary antibodies used were either anti-His (left) or anti-GST (right). Lane M, protein marker. Lane 1, His-VP2 protein. Lane 2, pET-28a. Lane 3, GST-VP2 protein. Lane 4, pGEX-4 T-1.

As shown in Figure 1, the purified His-VP2 and GST-VP2 were visualised on a PAGE gel after Coomassie Brilliant Blue staining. The purity of the recombinant protein was more than 90%. Furthermore, the Western blotting also showed that specific single bands appeared at 37 kDa and 52 kDa, respectively. These results indicate that recombinant VP2 proteins are successfully expressed and purified, which can be used as an immunogen for mouse immunisation, and also for subsequent monoclonal antibody preparation and epitope identification. In addition, pET-28a showed no detectable bands, whereas pGEX-4 T-1 produced a distinct band at 26 kDa, indicating that the GST tag expression was successful.

### 3.2 Screening and characterization of monoclonal antibodies

**Figure 2:**
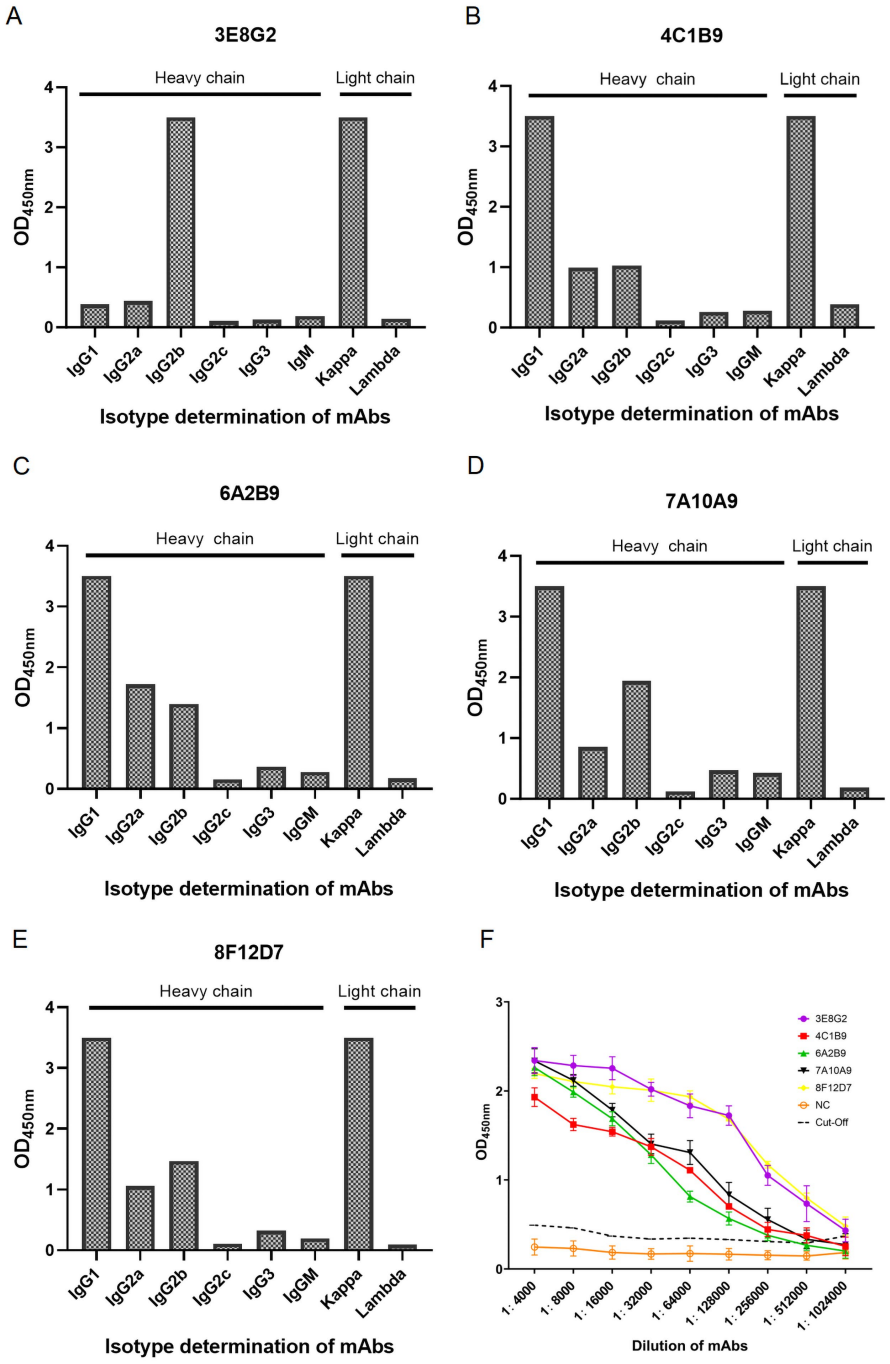
Subtype identification and titer of monoclonal antibodies produced. **(A)** Heavy and light chain isotypes of 3E8G2. **(B)** Heavy and light chain isotypes of 4C1B9. **(C)** Heavy and light chain isotypes of 6A2B9. **(D)** Heavy and light chain isotypes of 7A10A9. **(E)** Heavy and light chain isotypes of 8F12D7. **(F)** Titers of 3E8G2, 4C1B9, 6A2B9, 7A10A9, and 8F12D7 mAbs. NC is a negative serum.

Through three rounds of subcloning, we successfully obtained five monoclonal antibodies against VP2 by indirect ELISA and IFA, designated 3E8G2, 4C1B9, 6A2B9, 7A10A9 and 8F12D7 (Figure 2). The heavy chain isotype of 3E8G2 is IgG2b, the heavy chain isotypes of the other four mAbs are IgG1, and the light chain isotypes of five mAbs are kappa, as identified by the Mouse-derived Antibody Subtyping ELISA Kit (Figures 2A–E). The titers of five mAbs were determined by indirect ELISA, with titers of 1:1,024,000 for 3E8G2 and 8F12D7, 1:512,000 for 4C1B9 and 7A10A9, and 1:256,000 for 6A2B9 (Figure 2F).

**Figure 3:**
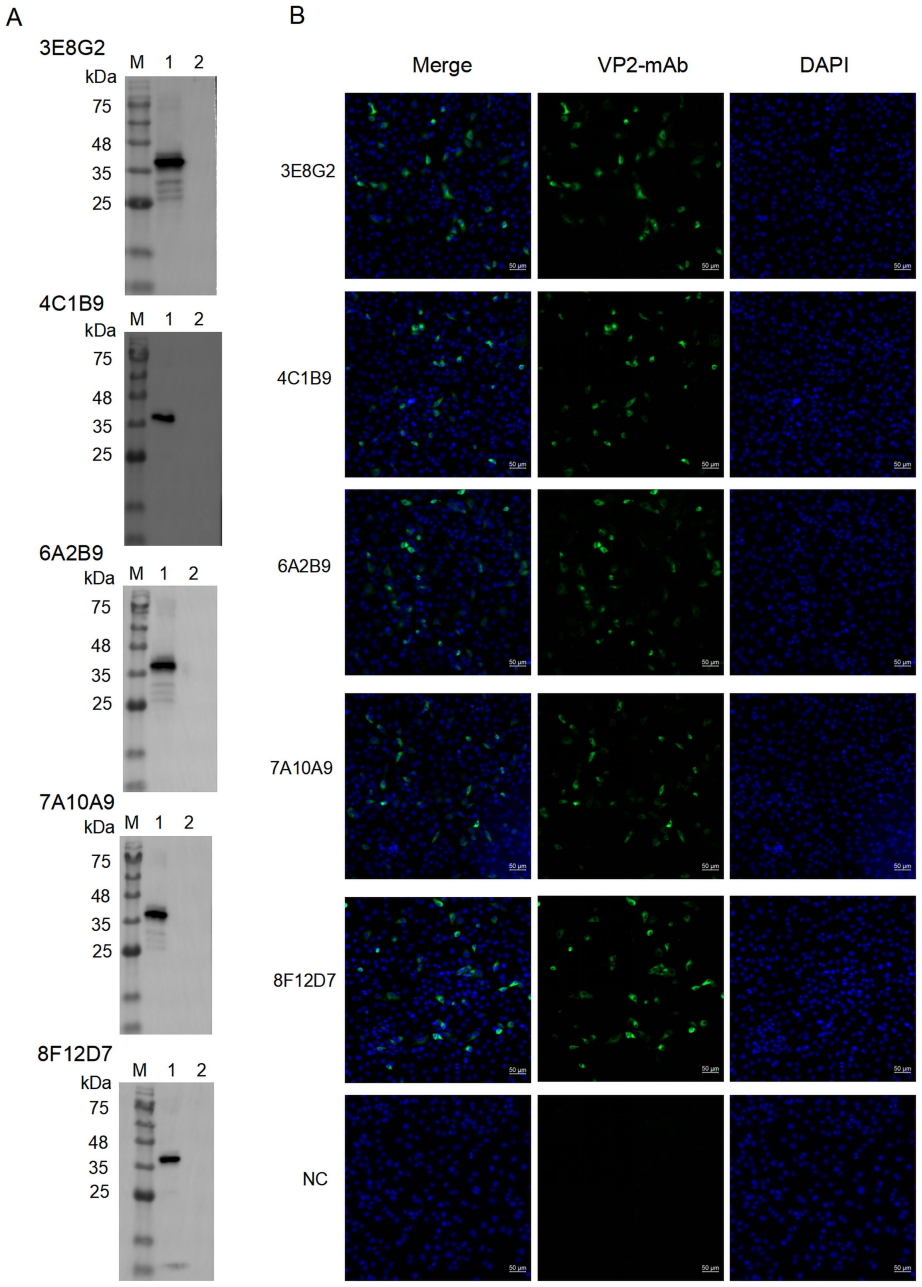
The reaction of the screened mAbs with His-VP2 protein was detected by Western blotting and IFA, respectively. **(A)** Western blotting assay for reactivity of mAbs 3E8G2, 4C1B9, 6A2B9, 7A10A9, and 8F12D7 with His-VP2 protein. Lane M, protein marker. Lane 1, His-VP2 protein. Lane 2, GST tag. **(B)** Binding of mAbs 3E8G2, 4C1B9, 6A2B9, 7A10A9, and 8F12D7 to SVA-infected PK-15 cells was detected by IFA. The cytopathic lesions are green and the nuclei are blue. The monoclonal antibody against the CD2V protein of African swine fever virus was used as a negative control. Scale bars, 50 μm.

Reactivity of the mAbs with VP2 protein was determined by Western blotting. As shown in Figure 3A, mAbs 3E8G2, 4C1B9, 6A2B9, 7A10A9 and 8F12D7 reacted specifically with His-VP2 protein, indicating recognition of linear epitopes on theVP2. IFA was conducted to assess the reactivity of mAbs with VP2 in SVA-infected cells. As shown in Figure 3B, the mAbs specifically bind to SVA-infected PK-15 cells, with reactivity localised in the cytoplasm. Thus, the mAbs specifically bind to VP2 in SVA-infected PK-15 cells.

### 3.3 Preliminary identification of B-cell epitopes of VP2 protein

**Figure 4:**
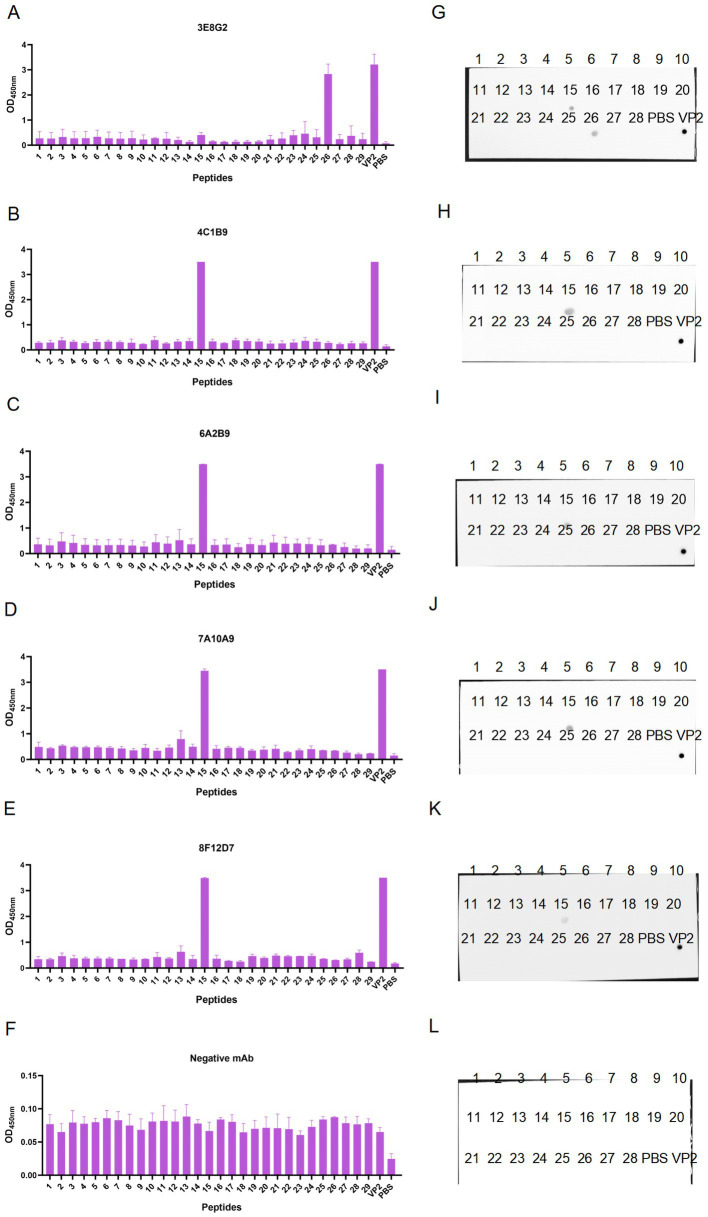
Preliminary identification of linear B-cell epitopes of VP2 protein using peptide scanning. **(A–E)** Recognition of 28 overlapping peptides with the prepared mAbs 3E8G2, 4C1B9, 6A2B9, 7A10A9, and 8F12D7 by peptide ELISA. **(F)** The monoclonal antibody against the CD2V protein of African swine fever virus was used as a negative control to recognise 28 overlapping peptides. **(G–K)** Recognition of 28 overlapping peptides by monoclonal antibodies was detected by Dot-blotting. VP2 protein was the positive control and PBS was the negative control. **(L)** Recognition of 28 overlapping peptides by the monoclonal antibody against the CD2V protein of African swine fever virus was assayed by Dot-blotting. VP2 protein was the positive control, PBS was the negative control.

**Figure 5:**
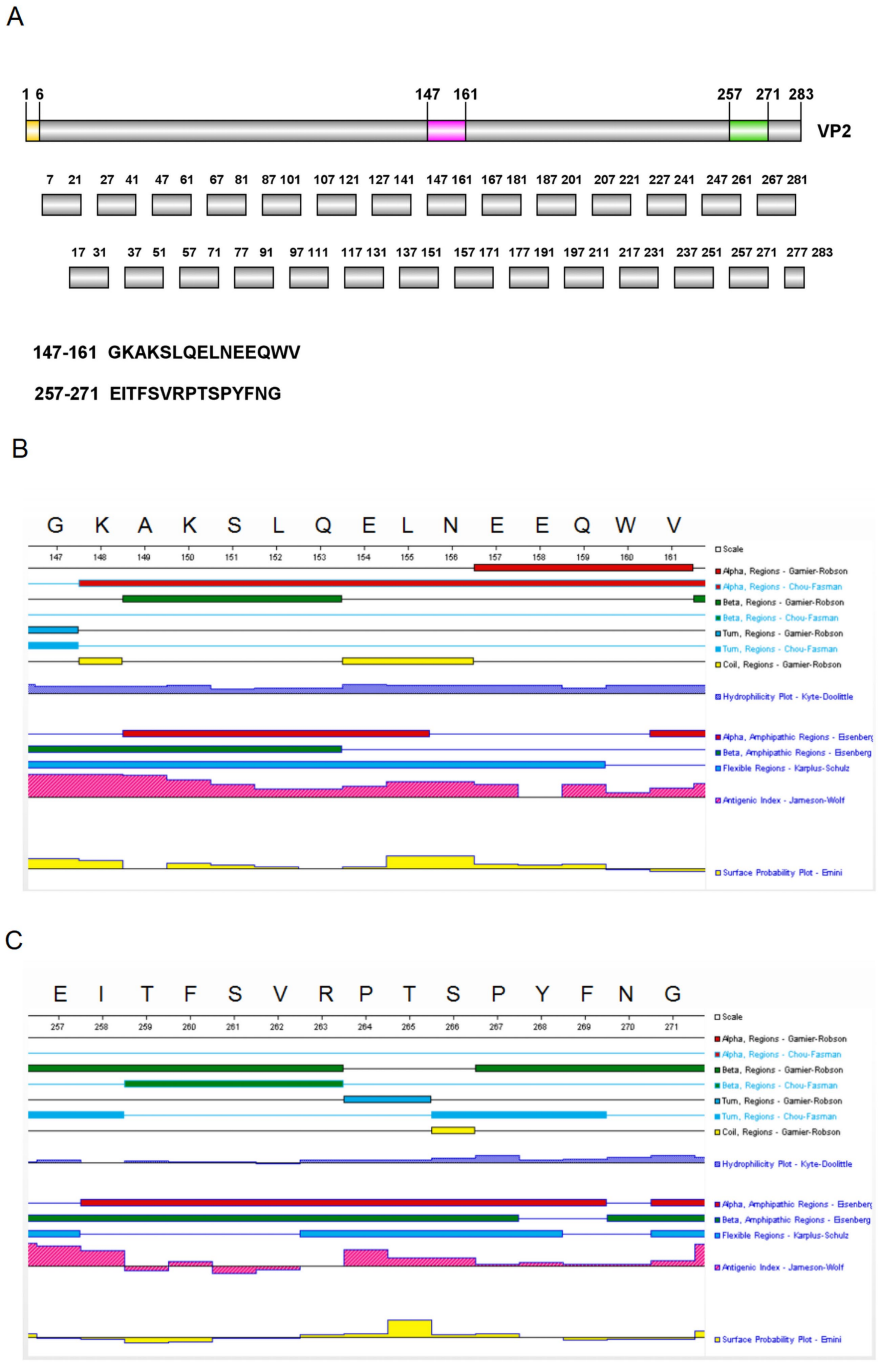
Peptides design and secondary structure analysis. **(A)** 28 short peptides were designed using the overlapping peptide synthesis. **(B)** The secondary structure of peptide 15 was predicted using DNAStar. **(C)** The secondary structure of peptide 26 was predicted using DNAStar.

As shown in Figure 4, the recognition of 28 peptides (Figure 5A) by five monoclonal antibodies was detected by Peptide ELISA and Dot-blotting. The mAb 3E8G2 reacted with peptides 15 and 26, while mAbs 4C1B9, 6A2B9, 7A10A9 and 8F12D7 reacted with peptide 15 (Figures 4A–F). The negative mAb did not react with any of the peptides. The mAbs against peptide 15 showed stronger reactivity with OD450nm values greater than 3. In addition, 28 peptides were also dotted on the NC membranes to analyse their reactivity with the five mAbs using Dot-blotting. 3E8G2 showed stronger reactivity with peptide 26, while the other mAbs specifically reacted with peptide 15 (Figures 4G–K). The negative mAb did not react with any of the peptides. The results of the peptide ELISA were in agreement with those obtained by Dot-blotting. In summary, two epitopes, 147-GKAKSLQELNEEQWV-161 and 257-EITFSVRPTSPYFNG-271, were initially identified by the five mAbs.

### 3.4 Identification of key amino acids for antigenic epitopes

**Figure 6:**
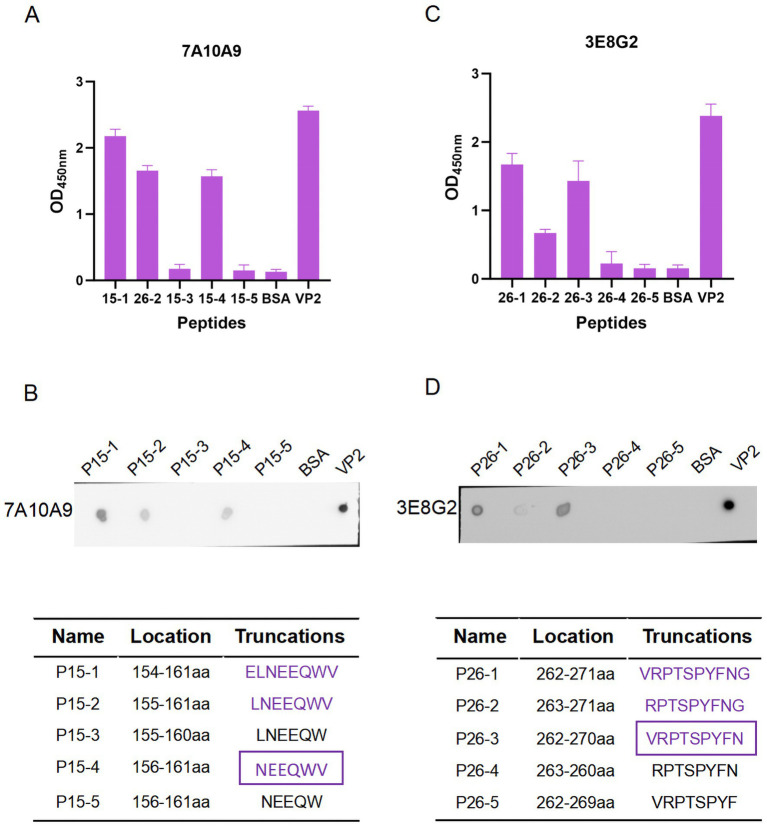
Minimal residue mapping of epitopes. **(A)** Recognition of short peptides P26-1, P26-2, P26-3, P26-4, and P26-5 with the mAb 3E8G2 by peptide ELISA. **(B)** Recognition of short peptides P26-1, P26-2, P26-3, P26-4, and P26-5 by mAb 3E8G2 was detected by Dot-blotting. **(C)** Recognition of short peptides P15-1, P15-2, P15-3, P15-4, and P15-5 with the mAb 7A10A9 by peptide ELISA. **(D)** Recognition of short peptides P15-1, P15-2, P15-3, P15-4, and P15-5 by mAb 7A10A9 was detected by Dot-blotting.

To determine the key amino acids in the epitopes, we analysed the antigenic epitope parameters (secondary structure, hydrophilicity, surface accessibility and antigenicity) of peptides 15 and 26 with DNAStar (Figures 5B,C). The regions with higher hydrophilicity, surface affinity and antigenicity as well as fewer *α*-helices and *β*-folds were selected to truncate peptide 15 into P15-1, P15-2, P15-3, P15-4 and P15-5, and peptide 26 into P26-1, P26-2, P26-3, P26-4 and P26-5 (Figure 6). Among them, P15-1, P15-2 and P15-4 were likely to react specifically with the five monoclonal antibodies (Figure 5B), while P26-1 and P26-2 were likely to react specifically with the five monoclonal antibodies (Figure 5C). Recognition of the short peptides with five monoclonal antibodies was detected by ELISA and Dot-blotting. The results showed that mAb 7A10A9 reacted specifically with P15-1, P15-2 and P15-4, while mAb 3E8G2 reacted with P26-1, P26-2 and P26-3. Neither antibody reacted with P15-3, P15-5, P26-4 or P26-5 (Figures 6A–D). These results confirmed the predictions. Overall, the key amino acid for the antigenic epitope recognised by mAb 3E8G2 on VP2 is 262-VRPTSPYFN-270 and the key amino acid for the epitope recognised by mAb 7A10A9 is 156-NEEQWV-161.

### 3.5 The genetic diversity and spatial distribution of the epitopes

**Figure 7:**
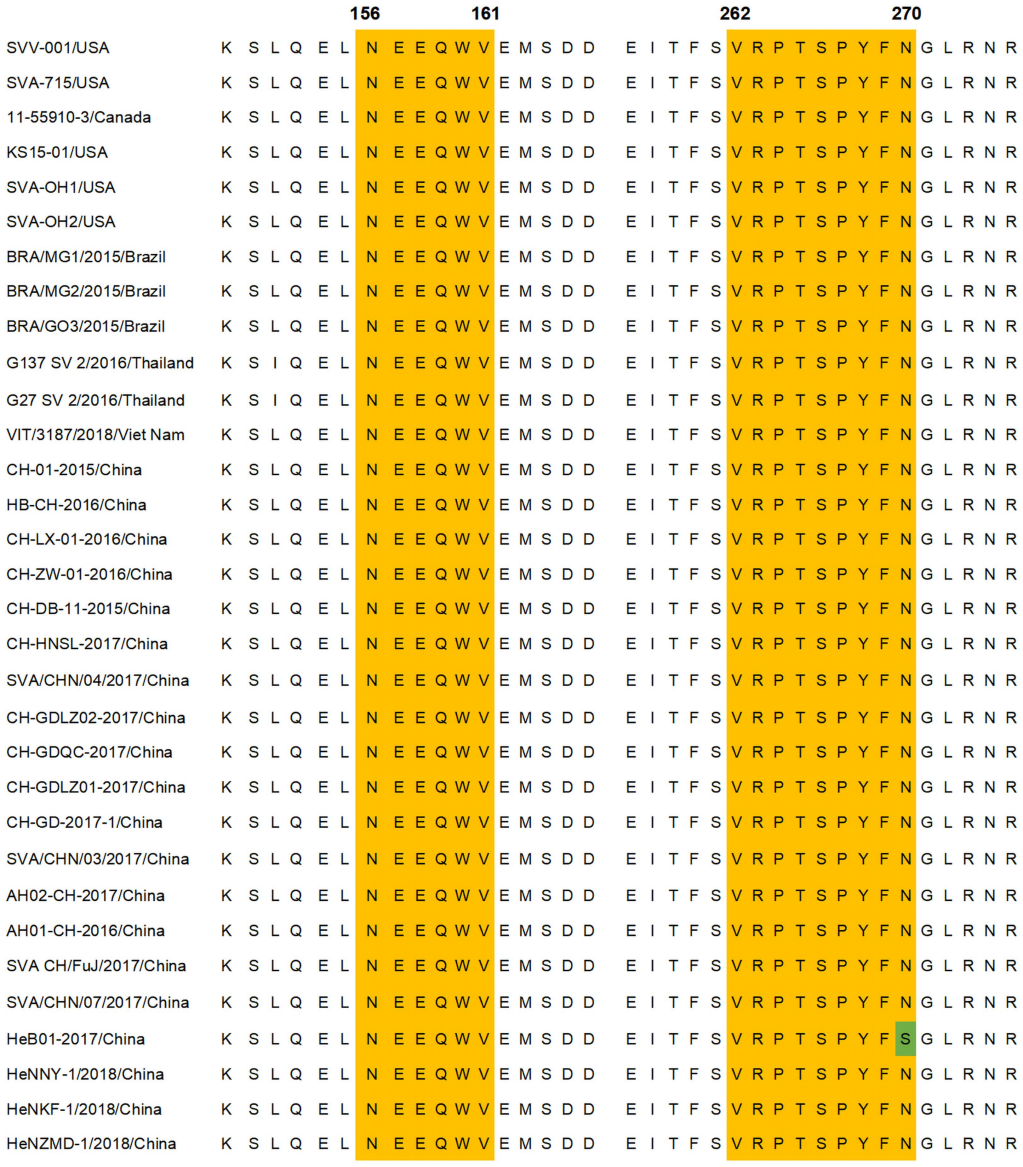
Amino acid sequence comparison of identified antigenic epitopes. Comparison of the amino acid sequences of VP2 protein epitopes in different SVA strains. The yellow area represents the epitope region, and the green area indicates the site of the amino acid mutation within the epitope region. Thirty-two VP2 protein sequences from SVA strains were obtained from GenBank.

Thirty-two representative SVA isolates from GenBank covering different countries were selected. The amino acid sequences of the 156-NEEQWV-161 and 262-VRPTSPYFN-270 regions were compared for epitope conservation. As shown in Figure 7, there was no amino acid variation within the 154-ELNEEQWV-161 region, while in the 262-VRPTSPYFN-270 region, the N270S mutation was found only in strain HeB01-2017/China, indicating a high conservation of these epitopes across strains.

**Figure 8:**
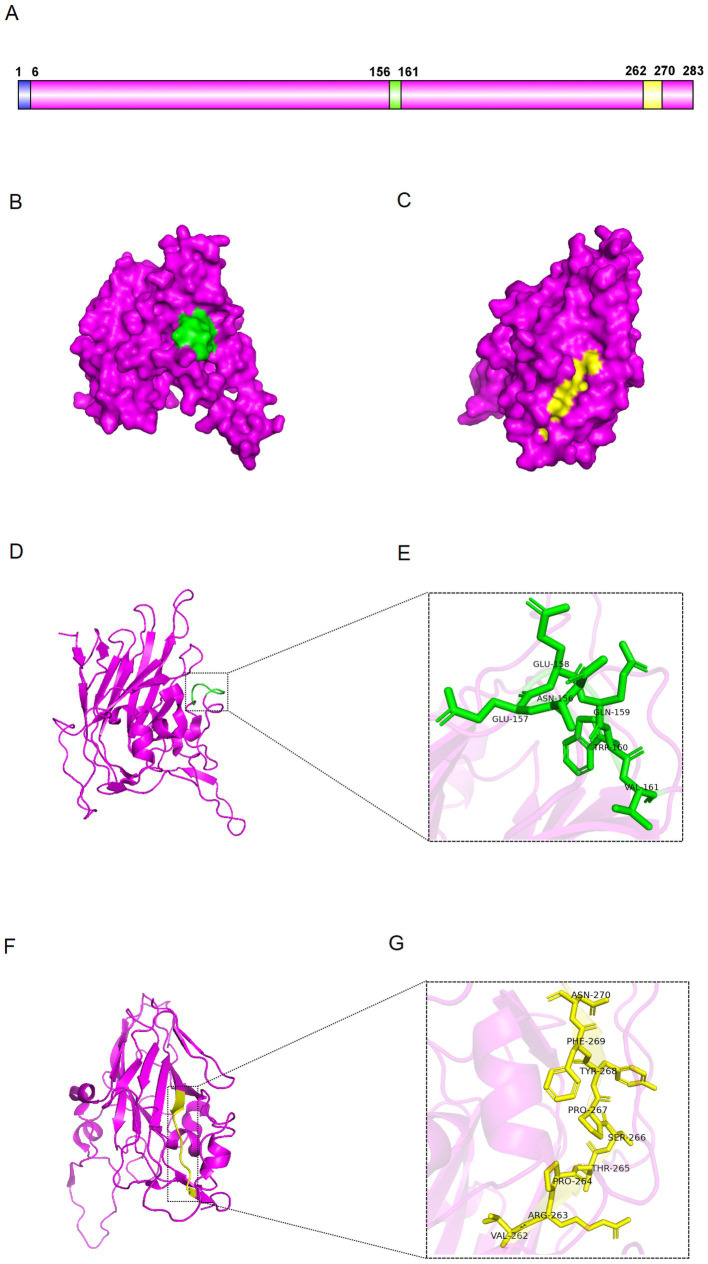
Distribution of the epitopes. **(A)** Schematic representation of VP2 protein. **(B,C)** Surface visualisation of VP2 protein. The green region represents epitope 156-NEEQWV-161, while the yellow represents epitope 262-VRPTSPYFN-270. **(D,E)** Cartoon view of VP2 protein. Enlarged view of the green region showing the amino acid sequence of epitope 156-NEEQWV-161. **(F,G)** Cartoon of VP2 protein. Magnified view of the yellow region showing the amino acid sequence of epitope 262-VRPTSPYFN-270.

To further analyse the epitopes, we used PyMOL to visualise the 3D structure of VP2 protein. Epitopes 156-NEEQWV-161 and 262-VRPTSPYFN-270 are exposed on the surface of VP2 protein (Figures 8A–C). The 156-NEEQWV-161 is located in the flexible loop region of the VP2, while the 262-VRPTSPYFN-270 is part of its *β*-sheet (Figures 8D,F). The regions of 156-NEEQWV-161 and 262-VRPTSPYFN-270 have been extended to mimic the epitope structure and show the spatial structure of key amino acid sites (Figures 8E,G). These results indicate that the three-dimensional structure of VP2 can expose key epitopes to stimulate active antibody production.

## 4 Discussion

As a novel animal infectious disease, the outbreak of Senecavirus A has caused significant economic losses to the global agricultural industry (Preis et al., 2022). Currently, there is no commercial vaccine against SVA, and no effective prevention strategies are available. Efforts are focused on laboratory diagnostics to enhance outbreak surveillance and control through source management, biosecurity and husbandry practices. Rapid field diagnostic methods and vaccine development are urgently needed. VP2 protein is highly immunogenic, inducing the body to produce potent, specific antibodies that play a critical role in long-term immune protection (Dvorak et al., 2017). This immunogenic property is essential for the development of effective SVA diagnostics and vaccines.

In this study, His-VP2 and GST-VP2 were expressed in an *E. coli* prokaryotic expression system. After purification, BALB/c mice were immunised with His-VP2 as the immunogen. GST-VP2 was used as the coated antigen for ELISA. After cell fusion, positive hybridoma cells were screened by ELISA and IFA. The combination of these two screening methods enables the mAbs with high specificity and sensitivity. In addition, the labelling of the immunoprotein is different from that of the coated protein in indirect ELISA, which helps to reduce cross-reactivity caused by labelling, thereby improving the success rate of monoclonal antibody screening. Finally, five hybridoma cell lines have been successfully obtained by subcloning.

The key to antigenic epitope prediction is to determine the protein’s structure and analyse the data to extract useful information. Immunoinformatics software supports the analysis of antigenic protein secondary structures and the prediction of antigenic epitopes through parameter-based comparisons. Alternatively, deep neural network algorithms are employed to predict antigenic epitope regions. In this study, 28 peptides of 15 amino acids each were synthesized by truncating the amino acid sequence of VP2 using peptide scanning. Subsequently, two positive peptides were identified through peptide ELISA and Dot-blotting. The secondary structure, hydrophilicity, surface affinity, and antigenicity of these two peptides were analysed using DNAStar. Among these parameters, secondary structure was deemed most critical, followed by hydrophilicity, while surface affinity had the least influence. Based on a combination of these parameters, the peptides were further truncated, and short peptides were re-screened using peptide ELISA and Dot-blotting to pinpoint the smallest functional region of the epitope. This classical method of epitope prediction, which relies on human comparison of various parameters, is highly subjective and can reduce the accuracy of predictions. By combining the experimental data with the prediction results and gradually updating the prediction method, the accuracy of the prediction will continue to improve.

Using PyMOL, the three-dimensional structure of the VP2 were visualised. The epitopes are exposed on the surface of the VP2 protein and does not interact with neighbouring proteins, making it an ideal immune target. According to previous reports, VP2 D146 interacts with metal ions in Anthrax Toxin Receptor 1 (ANTXR1) and is required for SVA entry into cells (Cao et al., 2018). VP2 147–161 is an antigenic epitope, with 156–161 being a key amino acid residue that determines antibody binding. The VP2 147–161 region is in close proximity to VP2 D146 and may indirectly regulate ANTXR1 binding by affecting the stability of the VP2 structure. Antibodies to VP2 147–161 may neutralise the virus by interfering with ANTXR1 binding or by altering the podoplanar structure, but the exact mechanism requires further investigation. This region may be a potential target for optimisation of SVA vaccines, either by enhancing immunogenicity to improve antibody responses, or by optimising antigenic epitopes to reduce immune escape and improve immune protection. The two minimal epitope regions were highly conserved by comparative sequence analysis. By targeting the epitopes, we can create specific, sensitive assays to assess SVA infection in pigs, helping prevent outbreaks and implement control measures. In addition, highly conserved epitopes could be valuable in vaccine design to ensure effectiveness against a wide range of virus strains.

While monoclonal antibodies are valuable tools for epitope research, only a few studies to date have accurately identified SVA epitopes. Zhang et al. used a combined bioinformatics and overlapping peptide screening approach to identify B-cell epitopes at positions 38–57, 145–160, 154–172, 193–208, and 249–284 on the VP2 protein, which were characterized with polyclonal antibodies (Zhang et al., 2023). Polyclonal antibodies can recognise several different epitopes on the same antigen. They are also able to recognise the antigen even when some epitopes are hidden or modified, making them more resilient to antigenic variation. However, polyclonal antibodies often exhibit significant batch-to-batch variability, which can affect experimental reproducibility. Fan et al. generated monoclonal antibodies using prokaryotically expressed VP2 protein and identified linear B-cell epitopes at positions 12–18, 71–76, 98–103, 150–156, and 248–253 (Fan et al., 2020). Monoclonal antibodies specifically bind to individual epitopes, reducing cross-reactivity and enhancing targeting accuracy in diagnostic applications. In this study, five monoclonal antibodies were produced using the hybridoma technique, with His-VP2 as the immunogen. VP2 was truncated and scanned with the prepared mAbs, and two positive peptides were identified. The positive peptides were further truncated based on DNAStar analysis, and the minimal epitope regions, 156-NEEQWV-161 and 262-VRPTSPYFN-270, were identified using the mAbs. These two linear epitopes have been identified for the first time using monoclonal antibodies. These findings enhance our understanding of VP2 structure, aid the development of SVA diagnostics and provide a basis for novel vaccine design.

## Data Availability

The datasets presented in this study can be found in online repositories. The names of the repository/repositories and accession number(s) can be found in the article/Supplementary material.

## Footnotes

^1^ http://www.rcsb.org/
