# Supplementary material for: Identification of linear B-cell epitopes of Senecavirus A VP2 protein using monoclonal antibodies
Source: Front Microbiol. 2025 Mar 5;16:1546925. doi: 10.3389/fmicb.2025.1546925 (PMC11919882; doi:10.3389/fmicb.2025.1546925)
Supplement: Supplementary file 1 [file Table_1.doc]

Supplemental Table S1: Sequence information in this study.

| No. | Strain name | Accession No. | Origin | Year |
| --- | --- | --- | --- | --- |
| 1 | SVV-001 | NC_011349 | USA | 2008 |
| 2 | SVA-715 | KY172968 | USA | 2014 |
| 3 | KS15-01 | KX019804 | USA | 2015 |
| 4 | SVA-OH2 | KU058183 | USA | 2015 |
| 5 | SVA-OH1 | KU058182 | USA | 2015 |
| 6 | 11-55910-3 | KC667560 | Canada | 2011 |
| 7 | SVA/BRA/GO3/2015 | KR063109 | Brazil | 2015 |
| 8 | SVA/BRA/MG2/2015 | KR063108 | Brazil | 2015 |
| 9 | SVA/BRA/MG1/2015 | KR063107 | Brazil | 2015 |
| 10 | G137_SV_2/2016/Thailand | MF416220 | Thailand | 2016 |
| 11 | G27_SV_2/2016/Thailand | MF416218 | Thailand | 2016 |
| 12 | SVA/VIT/3187/2018 | MH704432 | Viet Nam | 2018 |
| 13 | CH-01-2015 | KT321458 | China,Guangdong | 2015 |
| 14 | SVA/HLJ/CHA/2016 | KY419132 | China,Heilongjiang | 2016 |
| 15 | CH/GXI09/2016 | KY038016 | China | 2016 |
| 16 | HB-CH-2016 | KX377924 | China,Hubei | 2016 |
| 17 | CH-GDYS02-2017 | MG428685 | China,Guangdong | 2017 |
| 18 | CH-GDYS01-2017 | MG428684 | China,Guangdong | 2017 |
| 19 | CH-GDYD-2017 | MG428683 | China,Guangdong | 2017 |
| 20 | CH-GDQC-2017 | MG428682 | China,Guangdong | 2017 |
| 21 | CH-GDLZ02-2017 | MG428681 | China,Guangdong | 2017 |
| 22 | CH-GDLZ01-2017 | MG428680 | China,Guangdong | 2017 |
| 23 | CH-HN-2017 | KY747511 | China,Henan | 2017 |
| 24 | CH-HNSL-2017 | KY747512 | China,Henan | 2017 |
| 25 | CH-FJ-2017 | KY747510 | China,Fujian | 2017 |
| 26 | CH-ZW-01-2016 | KX751946 | China,Guangdong | 2016 |
| 27 | CH-LX-01-2016 | KX751945 | China,Guangdong | 2016 |
| 28 | CH-DL-01-2016 | KX751944 | China,Guangdong | 2016 |
| 29 | CH-DB-11-2015 | KX751943 | China,Guangdong | 2015 |
| 30 | CH-GD-2017-2 | MF189001 | China,Guangdong | 2017 |
| 31 | CH-GD-2017-1 | MF189000 | China,Guangdong | 2017 |
| 32 | SVA CH/FuJ/2017 | MH490944 | China,Fujian | 2017 |
| 33 | SVA/CHN/17/2017 | MG765566 | China,Guangdong | 2017 |
| 34 | SVA/CHN/16/2017 | MG765565 | China,Guangdong | 2017 |
| 35 | SVA/CHN/15/2017 | MG765564 | China,Guangdong | 2017 |
| 36 | SVA/CHN/14/2017 | MG765563 | China,Guangdong | 2017 |
| 37 | SVA/CHN/13/2017 | MG765562 | China,Guangdong | 2017 |
| 38 | SVA/CHN/12/2017 | MG765561 | China,Guangdong | 2017 |
| 39 | SVA/CHN/11/2017 | MG765560 | China,Guangdong | 2017 |
| 40 | SVA/CHN/10/2017 | MG765559 | China,Guangdong | 2017 |
| 41 | SVA/CHN/09/2017 | MG765558 | China,Guangdong | 2017 |
| 42 | SVA/CHN/08/2017 | MG765557 | China,Guangdong | 2017 |
| 43 | SVA/CHN/07/2017 | MG765556 | China,Guangdong | 2017 |
| 44 | SVA/CHN/06/2017 | MG765555 | China,Guangdong | 2017 |
| 45 | SVA/CHN/05/2017 | MG765554 | China,Guangdong | 2017 |
| 46 | SVA/CHN/04/2017 | MG765553 | China,Guangdong | 2017 |
| 47 | SVA/CHN/03/2017 | MG765552 | China,Guangdong | 2017 |
| 48 | SVA/CHN/02/2017 | MG765551 | China,Guangdong | 2017 |
| 49 | SVA/CHN/01/2017 | MG765550 | China,Guangdong | 2017 |
| 50 | HeB01-2017 | MF967574 | China | 2017 |
| 51 | AH02-CH-2017 | MF460449 | China | 2017 |
| 52 | AH01-CH-2016 | MF460448 | China | 2016 |
| 53 | CH-04-2015 | KX173340 | China,Guangdong | 2015 |
| 54 | CH-02-2015 | KX173339 | China,Guangdong | 2015 |
| 55 | CH-03-2015 | KX173338 | China,Guangdong | 2015 |
| 56 | HeNZMD-1/2018 | MK357115 | China,Henan | 2018 |
| 57 | HeNNY-1/2018 | MK357116 | China,Henan | 2018 |
| 58 | HeNKF-1/2018 | MK357117 | China,Henan | 2018 |

The isolated strains were in bold.
